# Transcriptome-Based Identification of *AP2/EREBP* Genes Regulating Cuticle Formation in Tree Peony ‘Bai Wang Shi Zi’

**DOI:** 10.3390/plants15121911

**Published:** 2026-06-20

**Authors:** Xu Li, Zhimin Huang, Conghao Hong, Youyi Zang, Yongjuan Jiao, Mengxue Xu, Meiyu Qiao, Yixin Liang, Hongbo Gao

**Affiliations:** 1 National Engineering Research Center of Tree Breeding and Ecological Restoration, State Key Laboratory of Efficient Production of Forest Resources, College of Biological Sciences and Technology, Beijing Forestry University, Beijing 100083, China; 2 National Peony Gene Bank, Luoyang Peony Industry Development Center, Luoyang 471002, China

**Keywords:** tree peony, petal development, AP2/EREBP, cuticular wax, cuticular lipid, PsSHN1, PsWRI3

## Abstract

Tree peony (*Paeonia suffruticosa* Andr.) is a traditional ornamental plant of high economic and cultural value, but its flower longevity is often limited by petal water loss. Cuticular wax serves as an essential barrier against non-stomatal water loss, and the AP2/EREBP (APETALA2/Ethylene-Responsive Element Binding Protein) transcription factor family is known to regulate wax biosynthesis. However, little information is available on the roles of AP2/EREBP genes in petal cuticle formation in tree peony. In this study, we performed transcriptome sequencing on petals of the tree peony cultivar ‘Bai Wang Shi Zi’ at three developmental stages (early, middle, and late). Using the assembled transcriptomic data, we identified 29 high-confidence AP2/EREBP family members, which were phylogenetically classified into AP2, ERF, and DREB subfamilies. Expression profiling revealed that 18 of these genes exhibited stage-specific expression patterns during petal development. Among them, two homologs of *Arabidopsis SHN1* (*SHINE 1*) and *WRI3* (*WRINKLED 3*), designated *PsSHN1* and *PsWRI3*, showed peak expression at the middle stage. By co-expression analysis and phylogenetic comparison, three downstream candidate genes were identified and named *PsCER2*, *PsKAS1*, and *PsLTPG1*, based on their homology with known wax-related genes. Dual-luciferase reporter assays indicated that PsSHN1 and PsWRI3 can activate the promoters of *PsCER2*, *PsKAS1*, and *PsLTPG1*, suggesting a possible cooperative regulation of cuticle formation. Collectively, our findings provide promising candidate genes for prolonging floral lifespan by improving petal cuticular wax accumulation, and lay a preliminary foundation for molecular breeding and quality improvement of tree peony and other ornamental flowers.

## 1. Introduction

Tree peony, a perennial deciduous shrub native to China, belongs to the genus *Paeonia* (family Paeoniaceae) [1]. As one of China’s ten traditional famous flowers, it is widely praised as the ‘king of flowers’ for its exceptional beauty and fragrance, and occupies an important position in both the domestic and international floriculture industry and cultural tradition [2,3]. Beyond its ornamental value, tree peony is highly valued for cut-flower production, garden landscaping, and potted display [4,5,6]. However, the life of tree peony flowers is shortened due to petal water loss, which severely constrains their ornamental quality and economic value [7,8,9]. Notably, cuticular wax acts as the primary protective barrier against non-stomatal water loss [10,11]. Cuticular wax deposition directly determines the ability of plants to maintain water balance, thereby contributing to drought stress tolerance and flower longevity [12,13].

Despite variation in chemical composition among species and even across organs of the same plant, all plant cuticles share two main components: cutin, a lipid polyester predominantly composed of C16 and C18 hydroxy fatty acids or diacids, and waxes—long-chain aliphatic compounds embedded within and deposited on the cuticle surface [14]. Chemically, plant cuticular waxes consist primarily of very-long-chain fatty acids (VLCFAs) and their derivatives, with carbon chain lengths typically ranging from 20 to 34 carbons [15]. Additionally, waxes of certain species may contain aldehydes, alkanes, ketones, primary and secondary alcohols, alkyl esters, and other minor components [16,17]. Biosynthetically, wax formation involves three key steps: the de novo synthesis of C16 and C18 fatty acids in plastids, their elongation to VLCFAs in the endoplasmic reticulum (ER), and the conversion of VLCFAs into diverse wax components via either the acyl-reduction or decarbonylation pathway [18,19]. Subsequently, newly synthesized wax components are transported intracellularly to the plasma membrane and exported via ABC transporters, traverse the cell wall, and finally are deposited on the plant surface [20,21,22]. These cuticular waxes play a critical role in determining the barrier properties of the plant surface, and their composition and abundance directly influence cuticular permeability and water retention capacity. In both lily and rose, petals/tepals show higher cuticular transpiration and water permeability than leaves, largely due to their shorter average aliphatic chain lengths. Wax coverage varies among cultivars and organs, and a positive correlation exists between total wax content and tolerance to post-harvest water-loss stress [23,24,25]. The environment can also affect wax content. For example, snapdragon flowers grown at cooler temperatures (18 °C/16 °C) exhibit slightly elevated wax loads throughout development compared to those grown at 25 °C/20 °C [26].

At the regulatory level, the AP2/EREBP transcription factor family represents one of the largest transcription factor families in plants. Based on domain architecture, its members are classified into five subfamilies: AP2 (two AP2 domains), RAV (one AP2 and one B3 domain), and ERF, DREB, and Soloist (each with one AP2 domain) [27,28,29]. Functionally, these transcription factors are widely involved in plant growth and development, stress responses and determination of floral organ identity [30,31,32,33]. Importantly, accumulating evidence identifies the AP2/EREBP transcription factor family as a critical molecular target regulating cuticle formation of leaves. Specifically, ERF subfamily members (e.g., SHN1) directly activate wax biosynthetic genes [34,35,36,37,38] and AP2 subfamily members (e.g., WRI3) regulate fatty acid precursor supply for cuticular lipid biosynthesis [39,40], both playing key roles in this process [41]. Although the roles of AP2/EREBP family members in cuticle formation are relatively well-established in other plant species (e.g., *Arabidopsis* [34], wheat [42], and rice [43]), little is known about their functions in flowering ornamental plants. Moreover, most investigations have focused on regulatory mechanisms in leaves [44] and fruit peels [45], whereas those operating in petal tissues remain largely unexplored. Given that the petal’s capacity to resist water loss is critical for maintaining ornamental quality and extending floral longevity [8,46,47], elucidating the regulatory mechanisms of AP2/EREBP transcription factors in petal cuticle formation would be of considerable significance for the breeding of *Paeonia suffruticosa* and other ornamental species.

In this work, the tree peony cultivar ‘Bai Wang Shi Zi’ was used for transcriptome sequencing at three key stages of petal development: early, middle, and late. Using the assembled transcripts from transcriptomic data, we screened 29 high-quality and reliable AP2/EREBP family members and further analyzed their phylogenetic relationships and conserved motifs. Furthermore, by combining expression profiling with interaction assays, we identified candidate genes involved in cuticle formation and validated the activation of the promoters of candidate genes by two transcription factors. Thus, our study identified important members of the AP2/EREBP transcription factor family involved in cuticle formation during petal development of peony ‘Bai Wang Shi Zi’, and preliminarily linked molecular regulation to the extension of flower longevity.

## 2. Results

### 2.1. Transcriptome Data Filtering and Gene Identification

**Figure 1:**
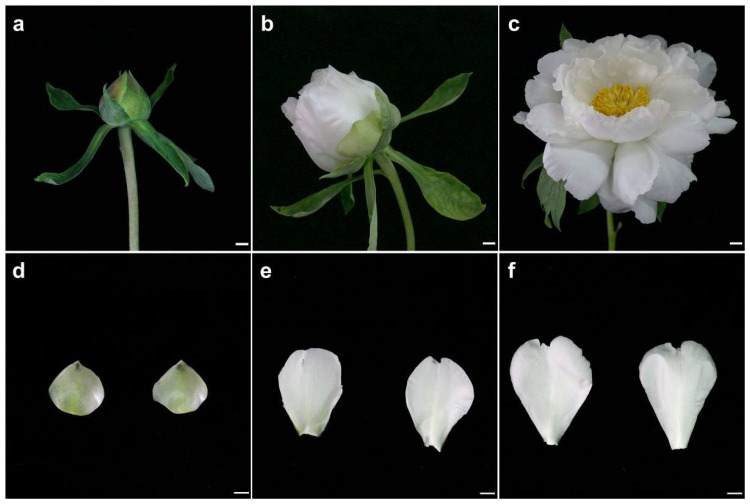
Three critical developmental phases of petals in ‘Bai Wang Shi Zi’. (**a**,**d**) Early phase: petals are fully enclosed by sepals. (**b**,**e**) Middle phase: petals are partially expanded. (**c**,**f**) Late phase: petals are fully expanded. Scale bars: 1 cm (applies to all figures).

Using the tree peony cultivar ‘Bai Wang Shi Zi’, we performed transcriptome sequencing at three key developmental stages of petals: early (A), middle (B), and late (C) (Figure 1a–c). Raw transcriptome reads were *de novo* assembled and translated into protein sequences, homology-based screening against the *Arabidopsis* AP2/EREBP family identified over 100 candidate AP2/EREBP transcription factors in ‘Bai Wang Shi Zi’. To ensure the reliability of subsequent analyses, stringent filtering criteria were applied: genes with very low expression at any developmental stage were removed, and genes with TPM < 1 were regarded as lowly or non-reliable and excluded. Ultimately, 29 high-confidence *AP2*/*EREBP* family genes were retained for subsequent bioinformatic and expression pattern analyses (Table 1).

### 2.2. Phylogenetic Analysis and Subfamily Classification

**Figure 2:**
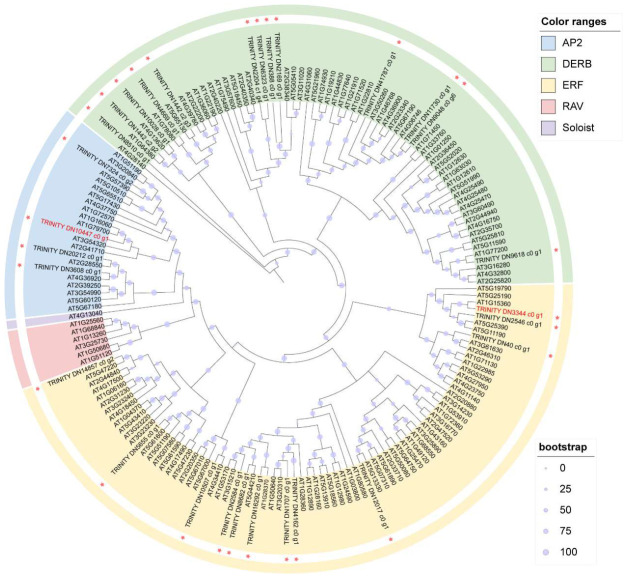
Phylogenetic tree of the *AP2*/*EREBP* gene family in *Paeonia suffruticosa* ‘Bai Wang Shi Zi’ and *Arabidopsis*. The tree was constructed based on protein sequences from *Arabidopsis* (147) and ‘Bai Wang Shi Zi’ (29). Different colors indicate different subfamily groupings. Gene IDs of ‘Bai Wang Shi Zi’ are marked with red stars. Bootstrap values are shown as purple circles at branch nodes, based on 1000 bootstrap replicates. Gene IDs labeled in red font are the target genes for subsequent studies.

To learn the diversification of *AP2*/*EREBP* gene family members in ‘Bai Wang Shi Zi’, a phylogenetic tree was constructed using 146 previously identified AP2/EREBP protein sequences from *Arabidopsis*. 29 corresponding protein sequences were identified in ‘Bai Wang Shi Zi’. The tree clearly divided all members into five subfamilies: AP2, ERF, DREB, RAV, and Soloist. All 29 tree peony proteins clustered within known subfamilies, and no independent branch was observed, indicating evolutionary conservation between *Arabidopsis* and peony. Specifically, four proteins belonged to the AP2 subfamily, 12 to the ERF subfamily, and 13 to the DREB subfamily, whereas no members of the RAV and Soloist subfamilies were detected in this study (Figure 2). This classification provides an evolutionary basis for subsequent functional analysis.

### 2.3. Analysis of Conserved Protein Domains and Motifs

**Figure 3:**
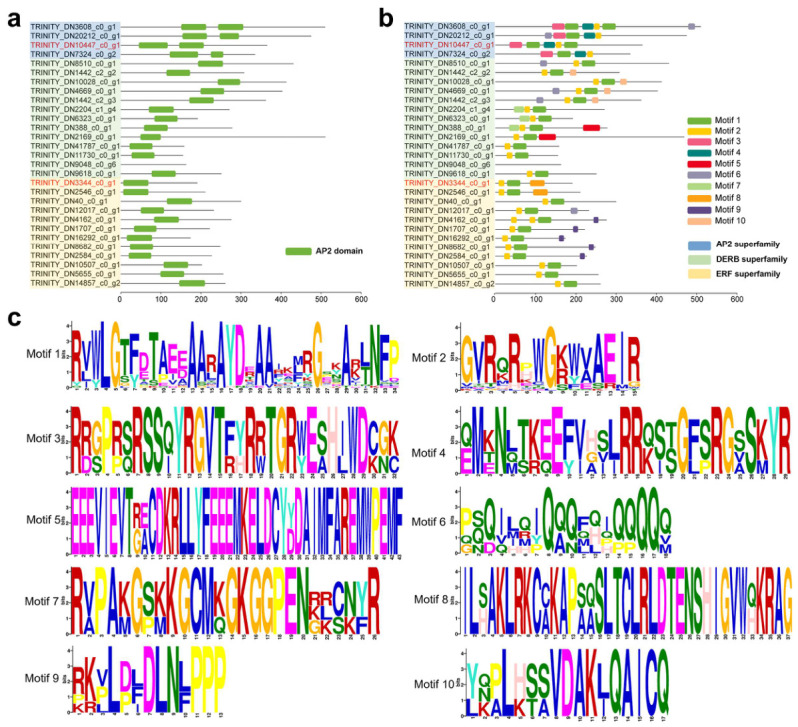
Analysis of conserved domains and motif composition of AP2/EREBP proteins in ‘Bai Wang Shi Zi’. (**a**) Distribution of conserved domains. Identified using the NCBI CDD database, colored bars represent the major domains: AP2 superfamily. (**b**) Composition of conserved motifs. Conserved motifs (motif 1–10) were predicted using MEME and are shown as colored rectangles, illustrating the specificity and diversity of motif combinations among members of different subfamilies. (**c**) Sequence logos of conserved motifs. The amino acid conservation of the 10 motifs shown in (**b**) is displayed, with letter height indicating the degree of conservation at each position.

Conserved domain analysis is essential for understanding the evolutionary relationships and functional divergence of gene subfamilies. To investigate the structural similarities and differences among these 29 AP2/EREBP proteins, we predicted their conserved domains using the NCBI Batch CD-Search tool. Conserved domain analysis showed that all proteins contained the canonical AP2 domain (Figure 3a). In addition to conserved domain characterization, the diversification of conserved motifs also contributes to the functional differentiation of transcription factor families. Analysis of protein motifs can provide insights into the regulatory mechanisms underlying TF-mediated transcription, protein interactions, and DNA recognition [48]. To identify shared motifs within each subfamily and distinct motifs among different subfamilies, we analyzed the protein sequences of these 29 members using the MEME (Multiple Em for Motif Elicitation) web server. MEME analysis identified ten conserved motifs, with their distribution patterns revealing marked subfamily specificity. The motif composition exhibited clear subfamily-specific patterns: the AP2 subfamily featured conserved motifs 1, 2, and 3; the ERF subfamily had motifs 1 and 2 as core motifs, often accompanied by motifs 3, 6, and 9; and the DREB subfamily showed more diverse and complex motif combinations (Figure 3b,c). Distinct motifs exist in each subfamily, acting as characteristic sequences for identifying and grouping members of the AP2/ERF superfamily and as determinants of the functional differences observed across subfamilies [49]. Furthermore, the conserved motifs within each subfamily may underlie shared functions among their members [50].

### 2.4. Expression Profile Analysis and Functional Prediction

**Figure 4:**
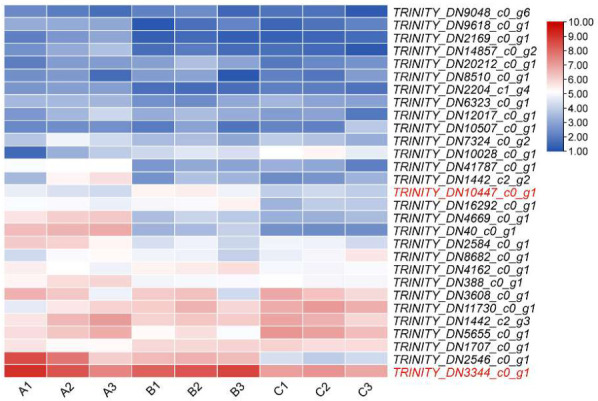
Expression patterns of the 29 *AP2*/*EREBP* genes during three petal developmental stages in ‘Bai Wang Shi Zi’. Heatmap was constructed based on TPM values of these genes at early (A), middle (B), and late (C) stages, with three biological replicates per stage. Color scale: blue (low expression) to red (high expression). Data were log_2_(TPM + 1)-transformed for all samples. Genes highlighted in red represent the candidates for further investigation.

By analyzing the expression patterns of 29 genes across the three stages of petal development, we classified them into two major groups: 11 genes showed low-level and stable expression; the remaining 18 genes exhibited distinct stage-specific expression patterns (Figure 4). For example, *TRINITY_DN11730_c0_g1* and *TRINITY_DN5655_c0_g1* were significantly up-regulated at the late stage (C), suggesting that they may positively regulate late petal development or senescence. Conversely, genes such as *TRINITY_DN4669_c0_g1* and *TRINITY_DN40_c0_g1* were down-regulated at the late stage, indicating potential functions in maintaining petal morphology and function during early developmental stages. Collectively, these 18 stage-specific genes were selected as key candidate genes for subsequent investigations into the molecular mechanisms underlying petal development.

**Figure 5:**
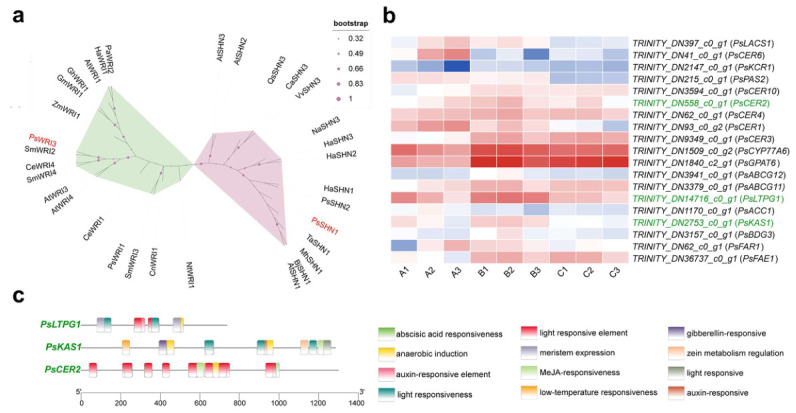
Phylogenetic, expression, and promoter analyses of candidate genes. (**a**) Phylogenetic tree of SHN and WRI proteins from various plant species. Including *Arabidopsis*, *Persea americana*, *Helianthus annuus*, *Nicotiana tabacum*, etc. (**b**) Transcriptome-based expression patterns of candidate genes involved in wax and fatty acid biosynthesis pathways. (**c**) Cis-acting element distribution in the promoters of *PsCER2*, *PsKAS1* and *PsLTPG1*. (Note: In panel (**a**), the proteins PsSHN1 and PsWRI3 are labeled in red font; in panels (**b**,**c**), PsCER2, PsKAS1, and PsLTPG1 are labeled with green font, respectively).

Among these genes, *TRINITY_DN3344_c0_g1* and *TRINITY_DN10447_c0_g1* from ‘Bai Wang Shi Zi’ were homologous to *AT1G15360* (*SHN1*) and *AT1G16060* (*WRI3*) in *Arabidopsis*, respectively. As shown in Supplementary Figure S1, both genes exhibited the highest expression at stage B, which is consistent with the expression trends of their *Arabidopsis* orthologs during petal development (flower developmental stage 12). Both SHN and WRI belong to the AP2/EREBP transcription factor family and contain AP2 domain. To clarify the differences in protein structure and evolutionary divergence between these two groups, we collected SHN and WRI family protein sequences from multiple species and constructed a phylogenetic tree. The results showed that SHN and WRI proteins were clearly clustered into two separate branches, indicating distinct evolutionary divergence (Figure 5a). Among them, PsSHN1 from the tree peony cultivar ‘Bai Wang Shi Zi’ was most closely related to TaSHN1 from *Triticum aestivum*, while PsWRI3 was most closely clustered with SmWRI2 from *Salvia miltiorrhiza*, revealing the respective species-specific evolutionary conservation of PsSHN1 and PsWRI3 in tree peony.

### 2.5. Screening and Identification of Candidate Target Genes of PsSHN1 and PsWRI3

Based on the transcriptome analysis above, two key genes, *PsSHN1* (*TRINITY_DN3344_c0_g1*) and *PsWRI3* (*TRINITY_DN10447_c0_g1*), were identified as core candidates for further investigation. To further investigate the target genes regulated by the two transcription factors, we analyzed the metabolic pathways (biosynthesis of waxes and fatty acids) potentially associated with each factor and subsequently screened for candidate functional genes. Transcriptome analysis revealed that *TRINITY_DN558_c0_g1*, *TRINITY_DN2753_c0_g1*, and *TRINITY_DN14716_c0_g1* exhibited peak expression at stage B, consistent with the expression patterns of the two transcription factors (Figure 5b). To explore the evolutionary relationships and interspecific homology of the above three genes and predict their potential functions, we constructed phylogenetic trees using their encoded proteins and homologous sequences from other species. The results revealed that PsCER2, PsLTPG1 and PsKAS1 shared the highest homology with AtSGR1 from *Arabidopsis*, QsLTPG1 from *Quercus suber* and VvKAS1_2 from *Vitis vinifera*, respectively (Supplementary Figure S2).

Previous studies have shown that SHN1 and WRI3 are AP2/EREBP family transcription factors that play important regulatory roles in cuticle biosynthesis: SHN1 activates wax biosynthetic genes [51], while WRI3 regulates fatty acid precursor supply for cuticle lipid synthesis [39]. Therefore, these three genes were selected as candidate target genes.

### 2.6. Promoter Architecture and Validation of Gene Expression Patterns

**Figure 6:**
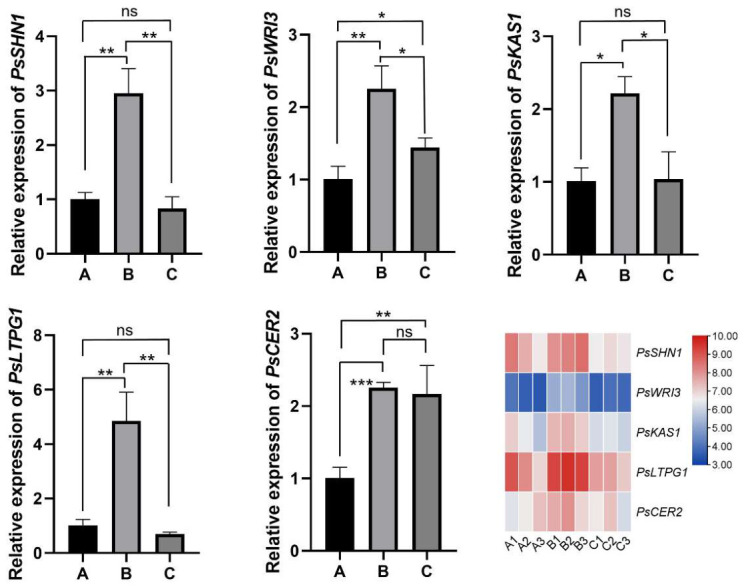
RT-qPCR analysis of candidate genes expression across three petal developmental stages (A, B, and C). Five genes were examined: *PsSHN1*, *PsWRI3*, *PsKAS1*, *PsLTPG1*, and *PsCER2*. *PsPP2AA3* served as the internal reference gene, with three biological replicates per stage. Error bars in panels represent mean + SD. Statistical significance is indicated as follows: * *p* ≤ 0.05; ** *p* ≤ 0.01; *** *p* ≤ 0.001; ns, not significant (*p* > 0.05) according to Tukey’s test. The heatmap (bottom right) displays temporal expression profiles across stages A–C, where red indicates high expression and blue indicates low expression.

To preliminarily explore the potential functional differences of the three candidate genes in metabolic regulation, we performed cis-acting element analysis on their promoter regions. The results revealed conservation and specificity among the promoters of *PsCER2*, *PsKAS1*, and *PsLTPG1*. All three promoters were rich in light-responsive and anaerobic induction elements, and generally contained multiple hormone-responsive elements, suggesting that their expression may be regulated by both environmental signals and endogenous hormones, implying functional associations (Figure 5c). However, the enrichment patterns of these elements differed among the genes: *PsCER2* was enriched with abscisic acid and jasmonate-responsive elements, indicating a tendency to participate in hormone-mediated stress and biosynthesis regulation; *PsKAS1* contained low-temperature, gibberellin, and metabolism-related regulatory elements, suggesting a broader range of signaling responses; whereas *PsLTPG1* had relatively fewer types of hormone-responsive elements and possessed a specific meristem expression element, potentially involved in meristem development regulation. These differences may reflect their functional divergence in distinct physiological processes. Following the promoter characteristic analysis, we further verified the expression trends of related genes at the transcriptional level. RT-qPCR was therefore applied to verify the RNA-seq results of the above five genes. The expression levels of all five genes peaked at stage B, consistent with the transcriptome data (Figure 6).

### 2.7. PsSHN1 and PsWRI3 Activate Important Target Genes Involved in Petal Cuticle Formation

To preliminarily explore whether PsSHN1 and PsWRI3 regulate the three candidate genes, we first performed bioinformatic predictions using the JASPAR 2026 (https://jaspar.elixir.no/analysis, accessed on 1 March 2026) database. Given the large genome size and incomplete annotation of tree peony, we used *Arabidopsis* AtSHN1 as a reference to predict its binding sites on target gene promoters. As shown in Supplementary Figure S2, the results showed that the *AtCER2* promoter (−242 to −1 bp) contains one potential binding site, whereas the *PsCER2* promoter (−1300 to −22 bp) of ‘Bai Wang Shi Zi’ was predicted to have six sites. The reason for selecting this *AtCER2* promoter region is that the NCBI (https://www.ncbi.nlm.nih.gov/) annotation indicates that at −242 bp upstream of the ATG of the *AtCER2* gene lies the UTR region of its upstream gene (Supplementary Figure S3). Similarly, the *AtLTPG1* and *PsLTPG1* promoters were predicted to contain three and seven binding sites, respectively (Supplementary Figure S4). These results provide preliminary bioinformatic clues that SHN1 may regulate *CER2* and *LTPG1*. Since the binding motif of WRI3 is currently not available in the JASPAR database, predicting its target sites by bioinformatics is not yet possible. However, previous studies have provided indirect evidence for a regulatory relationship between WRI3 and KAS1: CpWRI3 from *Carica papaya* L. was shown to specifically bind the AW-box element in the *CpKAS1* promoter, as validated by EMSA [52] and its ortholog WRI1 in *Arabidopsis* was also reported to directly bind the *KAS1* promoter [53]. However, in this study, no canonical motifs such as the AW-box were identified in the *PsKAS1* and *PsLTPG1* promoter regions using PlantCARE (https://bioinformatics.psb.ugent.be/webtools/plantcare/html/, accessed on 20 March 2026). This discrepancy may be attributable to species-specific variation, limitations in genome annotation, or constraints of the prediction method. Nevertheless, the above studies still provide indirect clues for a possible regulatory relationship between WRI3 and *KAS1*.

**Figure 7:**
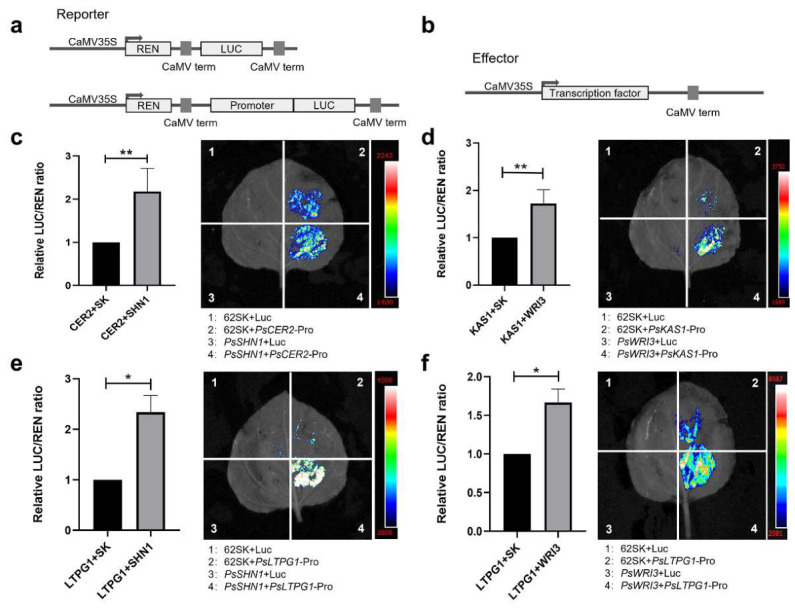
Verification of transcription factor-promoter interactions. (**a**) Schematic diagram of reporter vector construction: the promoters of target genes (*PsCER2*, *PsKAS1*, and *PsLTPG1*) were cloned into the pGreenII 0800-Luc vector, respectively. (**b**) Schematic diagram of effector vector construction: the encoding genes of transcription factors (PsSHN1 and PsWRI3) were cloned into the pGreenII 62-SK vector, respectively. (**c**) Dual-luciferase assays showed that PsSHN1 activated the *PsCER2* promoter, suggesting positive regulation of *PsCER2* transcription by PsSHN1. (**d**) Dual-luciferase assays showed that PsWRI3 activated the *PsKAS1* promoter, suggesting positive regulation of *PsKAS1* transcription by PsWRI3. (**e**) PsSHN1 activated the *PsLTPG1* promoter. (**f**) PsWRI3 activated the *PsLTPG1* promoter. Error bars indicate standard deviation (SD) of three independent replicates. Statistical significance was determined by Student’s *t*-test (* *p* < 0.05, ** *p* < 0.01).

Next, we constructed the vectors and conducted dual-luciferase reporter assays to investigate whether these two transcription factors activate or inhibit the promoter activity of the three candidate genes (Figure 7a,b). As shown in Figure 7c–f, the assays showed that PsSHN1 activates the wax biosynthetic gene *PsCER2*, and PsWRI3 activates the fatty acid synthesis gene *PsKAS1*, while both transcription factors also activate *PsLTPG1* (a lipid transfer protein gene involved in wax secretion) [54]. These findings suggest that PsSHN1 and PsWRI3 may cooperatively regulate petal cuticle formation in ‘Bai Wang Shi Zi’ through distinct but interconnected pathways.

## 3. Discussion

The cuticle, a hydrophobic protective structure covering the aerial surfaces of land plants and comprising cutin and cuticular wax [55,56], serves as a critical barrier that reduces non-stomatal water loss, prevents pathogen invasion, and mitigates UV and mechanical damage, thereby enhancing survival under adverse conditions [57,58,59,60]. These protective functions are especially vital in petals, where cuticular wax maintains water balance and withstands environmental stress, directly determining the ornamental quality and life of flowers [61,62,63].

The AP2/EREBP transcription factor family, one of the largest in plants, is widely involved in growth, development, stress responses, and cuticle formation [64,65,66]. This family serves as a key molecular target regulating the biosynthesis of this protective barrier [41], and its functional conservation has been demonstrated across diverse species. In *Arabidopsis*, *SHN1* overexpression increases total leaf wax sixfold and alkane content ninefold, while enhancing drought tolerance [38]. In maize, ZmEREB46 directly activates wax synthesis genes (e.g., *ZmCER2*); loss of this factor reduces leaf wax by ~50% and impairs drought tolerance [67]. In apple (*Malus domestica*), *MdSHN2* improves fruit gloss and water retention by regulating wax deposition [68], and MdWRI4 promotes wax biosynthesis and reduces epidermal permeability [69]. In *Brassica napus*, BnSHN1 enhances wax, oil, and osmotic tolerance without compromising yield [37]. SlSHN1 in tomato (*Solanum lycopersicum*) and TaSHN1 in wheat (*Triticum aestivum*) similarly boost wax accumulation and drought tolerance [42,70]. Collectively, these studies indicate that the AP2/EREBP family is functionally conserved in cuticle formation and stress adaptation across diverse plant species. However, its specific roles in petal cuticle development remain unclear. To elucidate this in tree peony, we identified AP2/EREBP family members in ‘Bai Wang Shi Zi’ via transcriptome sequencing and, through bioinformatic analyses, screened candidate genes involved in petal cuticle formation.

Through transcriptome assembly and screening, 29 high-quality AP2/EREBP genes were identified from ‘Bai Wang Shi Zi’ petals across three developmental stages. Subfamily classification revealed that ERF (12) and DREB (13) dominated, while only four belonged to AP2, and no RAV or Soloist members were detected, consistent with the predominance of ERF/DREB reported in most plants [71]. ERF/DREB members are mainly involved in stress responses and secondary metabolism [29,72,73], and specific members (e.g., ERF: SHN1 [37]; AP2: WRI3 [40]) are known regulators of cuticle formation. Conserved motif analysis showed subfamily-specific combinations (e.g., AP2 with motifs 1,2,3; ERF centered on motifs 1,2) supporting phylogenetic classification and reflecting functional domains including the AP2 DNA-binding domain and motifs potentially involved in protein-protein interactions [74,75]. RNA-seq revealed that 18 of the 29 AP2/EREBP genes exhibited stage-specific expression during tree peony petal development. Among these, *PsSHN1* and *PsWRI3* (homologs of *Arabidopsis SHN1* and *WRI3*) peaked at the middle stage (stage B), consistent with their *Arabidopsis* expression patterns. qRT-PCR validation of five selected genes confirmed the reliability of the RNA-seq data.

As important members of the AP2/EREBP family, SHN1 and WRI3 are known regulators of cuticular wax biosynthesis and fatty acid precursor supply, respectively [34,39,51]. Our dual-luciferase assays confirmed that PsSHN1 and PsWRI3 activate *PsCER2*, *PsKAS1*, and *PsLTPG1* in ‘Bai Wang Shi Zi’ (as shown in Results). Previous studies have revealed that *CER2* participates in VLCFA elongation during wax synthesis [76,77,78], *KAS1* drives de novo fatty acid production [79], and *LTPG1* mediates cuticular lipid transport [54,80]. Our experiments have demonstrated these potential regulatory relationships, which are similar to those reported for SHN1 in the leaves of *Arabidopsis*, maize, and tomato leaves [38,67,70], as well as for WRI3 in *Arabidopsis* seeds [39], suggesting that the functional roles of these AP2/EREBP members in cuticle formation may be evolutionarily conserved. However, this study remains preliminary, and the aforementioned regulatory relationships require further validation through genetic transformation in ‘Bai Wang Shi Zi’, yeast one-hybrid assays, or chromatin immunoprecipitation (ChIP). In conclusion, this finding provides a theoretical basis and candidate targets for subsequent in-depth elucidation of the molecular regulatory network of PsSHN1 and PsWRI3 in cuticle formation in *Paeonia suffruticosa* petals.

Petal senescence is essentially a specialized PCD process, and multiple exogenous and endogenous signals act as key triggers to initiate premature PCD in floral tissues, leading to accelerated petal withering and shortened ornamental lifespan [81,82]. Under natural growth conditions, petal epidermal cells are vulnerable to continuous water deficit, oxidative damage from UV radiation and high temperature, as well as invasion of pathogenic microorganisms. These adverse stresses can induce excessive accumulation of reactive oxygen species (ROS) in petal cells, disrupt cellular osmotic balance and membrane stability, and ultimately enhance the PCD signaling cascade to trigger premature floral senescence [83,84]. Petal cuticular wax accumulation has been reported to be closely correlated with improved flower longevity. As a protective physical barrier, cuticular wax can mitigate non-stomatal water loss, sustain cellular turgor, and defend against pathogen invasion, which may collectively delay petal senescence and prolong ornamental lifespan. For instance, Pearson correlation analysis in rose demonstrated a positive relationship between petal total wax content and flower longevity [12]; in tulip, the TgFbox1-TgNAC2-TgWIN1 regulatory module has been shown to fine-tune wax biosynthesis and alleviate petal senescence [85]. Collectively, exploring the molecular regulatory mechanisms of petal cuticle formation may provide important insights for theoretical research and molecular breeding in the floriculture industry. In future studies, it would be worthwhile to overexpress key wax regulatory genes, such as PsSHN1 and PsWRI3, through genetic manipulation, to increase petal wax abundance and optimize wax composition. From a translational perspective, this study provides a tentative but promising strategy for future floral longevity improvement in tree peony. Targeted elevation of petal epidermal wax deposition may serve as a feasible approach to alleviate stress-induced premature PCD and potentially extend floral lifespan.

## 4. Materials and Methods

### 4.1. RNA Extraction and Transcriptome Sequencing

The tree peony cultivar ‘Bai Wang Shi Zi’ was collected from the National Peony Garden in Luoyang. Petals were sampled at three key developmental stages: early (A, bell stage), middle (B, color-emergence stage), and late (C, full-bloom stage), with three biological replicates for each stage. Total RNA was extracted using the Tiangen RNAprep Pure Plant Kit (TIANGEN BIOTECH, Beijing, China) according to the manufacturer’s protocol. High-quality RNA samples were sequenced on an Illumina NovaSeq 6000 platform (Novogene, Beijing, China).

### 4.2. RNA-Seq Data Assembly and Analysis

Paired-end RNA-seq reads were assembled using Trinity v2.15.2 [86] under the Ubuntu 22.04 LTS system on an Intel Core i9-10900K server. The assembly was performed with 20 CPU threads and a maximum memory allocation of 120 GB. All other parameters were kept at the default settings. The assembled transcript sequences were used for subsequent transcriptome analysis. Gene expression levels were quantified as TPM values to correct for gene length and sequencing depth, enabling reliable comparison among samples.

### 4.3. Phylogenetic Analysis of AP2/EREBP Proteins

The AP2/EREBP protein sequences of *Arabidopsis* were retrieved from PlantTFDB 5.0 (https://planttfdb.gao-lab.org/). Multiple sequence alignment and phylogenetic analysis were performed using IQ-TREE multicore version 2.0.7, comparing *Arabidopsis* proteins with 29 AP2/EREBP proteins identified in ‘Bai Wang Shi Zi’. The phylogenetic tree was constructed using the maximum-likelihood (ML) method with 1000 bootstrap replicates and subsequently visualized using the online tool iTOL (https://itol.embl.de/). The reference genomic data was obtained from *P. ostii* [87].

### 4.4. Motif Analysis of AP2/EREBP Genes

Conserved domains were identified using NCBI Batch CD-Search (https://www.ncbi.nlm.nih.gov/Structure/cdd/wrpsb.cgi, accessed on 1 February 2026) with default parameters (E-value ≤ 0.01). Conserved motifs were predicted using the MEME Suite online server (https://meme-suite.org/meme/, accessed on 5 February 2026) with a maximum of 10 motifs. Integrated visualization of gene structures and conserved motifs was constructed using TBtools-II (v2.390) [88].

### 4.5. Cloning of Promoters and Coding Sequences from ‘Bai Wang Shi Zi’

To obtain promoter sequences of the three genes, primers were designed based on the *P. ostii* genome [87], a closely related cultivar with high sequence similarity. Based on the *P. ostii* genome sequence, upstream and downstream primers were designed at positions −1300 bp and −22 bp for *PsCER2*, −1285 bp and −19 bp for *PsKAS1*, and −736 bp and −1 bp for *PsLTPG1* relative to the respective ATG codons. The upstream sequences of these sequences are mostly repeats. So, they were avoided for PCR amplification and the constructions. For detailed information on the primers, please refer to Supplementary Table S3. Following PCR amplification, the products were Sanger-sequenced, and the authentic promoter sequences of the three genes in “Bai Wang Shi Zi” were obtained (Supplementary Figures S5–S7). Similarly, we obtained the coding sequences of the *SHN1* and *WRI3* genes from the tree peony cultivar ‘Bai Wang Shi Zi’. The coding sequence length of *PsSHN1* is 587 bp, and that of *PsWRI3* is 1095 bp. The detailed sequences and their alignment with *P. ostii* are presented in Supplementary Figures S8 and S9.

### 4.6. Dual-Luciferase Reporter Assay

The promoter sequences of the three genes were cloned into the pGreenII 0800-LUC vector [89] to generate reporter constructs, whereas the coding sequences of the other two genes were cloned into the pGreenII 62-SK vector to generate effector constructs. The empty vectors pGreenII 62-SK and pGreenII 0800-LUC, as well as the constructed recombinant vectors, were separately transformed into *Agrobacterium tumefaciens* GV3101 (pSoup-p19) competent cells. Bacterial cultures were grown to OD600 = 0.4, pelleted, and resuspended in infiltration buffer. The effector vector carrying the transcription factor gene (pGreenII 62-X) served as the test group, and the empty effector vector (pGreenII 62-SK) served as the control group. The reporter vector and each effector vector were mixed at a 1:1 volume ratio as Agrobacterium suspensions.

Suspensions were infiltrated into leaves of 6-week-old *Nicotiana benthamiana*. Each leaf was divided into four infiltration zones, with each zone receiving a different bacterial mixture. In different experimental groups, sites and areas of tobacco leaf infiltration and Agrobacterium usage were kept as consistent as possible. After infiltration, plants were incubated at 22–25 °C in darkness for 48 h. Luciferase activity was detected by spraying luciferase substrate onto the infiltrated areas and incubating in the dark for 10 min. Luminescence images were captured using a cooled CCD imaging system. Dual-luciferase assays were performed to detect luminescence activity. The LUC/REN ratio was calculated after normalization to REN activity. Using the empty vector pGreenII 62-SK as control, the transcriptional regulation pattern of the transcription factor on target genes was determined. All experiments included three biological replicates.

### 4.7. Determination of Dual-Luciferase Activity

Luciferase activity was assayed at 48 h after agroinfiltration using the Firefly & Renilla Dual Luciferase Assay Kit (Biorigin, Beijing, China) according to the manufacturer’s instructions. Firefly and Renilla luminescence were sequentially measured using a luminometer, and relative activity was expressed as the Firefly/Renilla ratio. Data from three independent biological replicates were analyzed using SPSS 26.0 and plotted using GraphPad Prism 8.0.

### 4.8. Quantitative Real-Time PCR (qPCR) Analysis

Total RNA was reverse-transcribed into cDNA using the M5 HiPer First Strand cDNA Synthesis Kit (Mei5bio, Beijing, China). Using cDNA as a template and *PsPP2AA3* as the reference gene, the expression levels of five target genes were detected by qPCR. The primer sequences are provided in Supplementary Table S1. The qPCR reactions were performed using SYBR Green reagent (Mei5bio, Beijing, China), and the reaction program is set out in Supplemental Materials (Table S2).

## 5. Conclusions

In this study, 29 AP2/EREBP family genes were systematically identified and characterized in tree peony ‘Bai Wang Shi Zi’ petals based on transcriptomic data across key developmental stages. Two core members, PsSHN1 and PsWRI3, were further functionally investigated. Dual-luciferase assays confirmed that both transcription factors positively activate the wax synthesis and transport genes *PsCER2*, *PsKAS1*, and *PsLTPG1*, suggesting their conserved roles in promoting petal cuticular wax deposition.

Notably, this work preliminarily reveals a potential regulatory association between AP2/EREBP-mediated wax accumulation and flower longevity. As petal senescence is a typical PCD-dominated process, increased wax deposition may mitigate environmental stress-induced oxidative and osmotic damage, inhibit premature PCD, and thereby delay petal senescence. These results provide preliminary mechanistic insights into petal cuticle formation and offer valuable candidate genes for floral longevity regulation in woody ornamental plants.

Nevertheless, this study remains preliminary, lacking in vivo genetic evidence and systematic verification of the crosstalk between wax metabolism and PCD-related senescence signaling. Future work will further validate the biological functions of PsSHN1 andPsWRI3, refine their regulatory networks in petal wax synthesis, and explore feasible strategies to improve floral lifespan by enhancing petal wax accumulation. Collectively, our findings lay a tentative foundation for the molecular breeding and quality improvement of tree peony and other floricultural crops.

## Figures and Tables

**Table 1:** Protein feature analysis of 29 candidate genes screened from transcriptome data of the tree peony cultivar ‘Bai Wang Shi Zi’.

Protein (Bai Wang Shi Zi)	Homolog in *P. ostii*	Identity (%)	Homolog in *Arabidopsis*	E Value	Length (aa)	SCL Prediction
TRINITY_DN3608_c0_g1	Pos.gene23053	99%	AT4G36920	1.00× 10^−136^	530	Nuclear
TRINITY_DN20212_c0_g1	Pos.gene44698	99%	AT2G28550	7.00 × 10^−118^	492	Nuclear
TRINITY_DN10447_c0_g1	Pos.gene38329	94%	AT1G16060	2.00 × 10^−116^	391	Chloroplast
TRINITY_DN7324_c0_g2	Pos.gene48543	100%	AT1G51190	5.00 × 10^−33^	149	Chloroplast
TRINITY_DN8510_c0_g1	Pos.gene19563	99%	AT2G20880	3.00 × 10^−61^	447	Nuclear
TRINITY_DN1442_c2_g2	Pos.gene38190	99%	AT1G64380	6.00 × 10^−72^	320	Nuclear
TRINITY_DN10028_c0_g1	Pos.gene62456	98%	AT4G13620	5.00 × 10^−76^	429	Nuclear
TRINITY_DN4669_c0_g1	Pos.gene30127	98%	AT1G78080	1.00 × 10^−94^	419	Nuclear
TRINITY_DN1442_c2_g3	Pos.gene19189	98%	AT3G50260	9.00 × 10^−25^	482	Endoplasmic reticulum
TRINITY_DN2204_c1_g4	Pos.gene27517	96%	AT2G40340	7.00 × 10^−48^	286	Nuclear
TRINITY_DN6323_c0_g1	Pos.gene46664	99%	AT2G40350	3.00 × 10^−52^	198	Nuclear
TRINITY_DN388_c0_g1	Pos.gene71548	86%	AT5G18450	5.00 × 10^−31^	404	Nuclear
TRINITY_DN2169_c0_g1	Pos.gene19315	84%	AT5G05410	5.00 × 10^−17^	798	Nuclear
TRINITY_DN41787_c0_g1	Pos.gene3622	100%	AT2G23340	2.00 × 10^−59^	125	Nuclear
TRINITY_DN11730_c0_g1	Pos.gene60806	100%	AT1G46768	2.00 × 10^−53^	282	Cytoplasmic
TRINITY_DN9048_c0_g6	Pos.gene63682	98%	AT2G38340	2.00 × 10^−39^	340	Nuclear
TRINITY_DN9618_c0_g1	Pos.gene36514	86%	AT5G11590	4.00 × 10^−57^	223	Nuclear
TRINITY_DN3344_c0_g1	Pos.gene55738	99%	AT1G15360	2.00 × 10^−82^	197	Chloroplast
TRINITY_DN2546_c0_g1	Pos.gene31556	99%	AT5G25390	8.00 × 10^−72^	219	Nuclear
TRINITY_DN40_c0_g1	Pos.gene13975	99%	AT1G71130	1.00 × 10^−28^	313	Nuclear
TRINITY_DN12017_c0_g1	Pos.gene43244	99%	AT5G61890	1.00 × 10^−48^	254	Nuclear
TRINITY_DN4162_c0_g1	Pos.gene5011	99%	AT1G50640	2.00 × 10^−62^	240	Nuclear
TRINITY_DN1707_c0_g1	Pos.gene4894	99%	AT3G20310	2.00 × 10^−40^	230	Nuclear
TRINITY_DN16292_c0_g1	Pos.gene16885	97%	AT1G28370	9.00 × 10^−42^	188	Nuclear
TRINITY_DN8682_c0_g1	Pos.gene47908	99%	AT5G44210	1.00 × 10^−41^	256	Nuclear
TRINITY_DN2584_c0_g1	Pos.gene15645	99%	AT3G15210	1.00 × 10^−46^	235	Nuclear
TRINITY_DN10507_c0_g1	Pos.gene76444	99%	AT4G34410	2.00 × 10^−42^	210	Chloroplast
TRINITY_DN5655_c0_g1	Pos.gene56182	100%	AT5G51190	8.00 × 10^−57^	266	Nuclear
TRINITY_DN14857_c0_g2	Pos.gene73001	98%	AT5G47220	2.00 × 10^−76^	272	Nuclear

Note: The table lists the amino acid sequence identity with the homologous protein from *P. ostii*, the BLAST+ 2.17.0 E-value against the *Arabidopsis* ortholog, the protein length (amino acids), and the predicted subcellular localization (SCL) of each protein by WoLF PSORT (https://wolfpsort.hgc.jp/ (accessed on 17 June 2026)).

## Data Availability

Data is contained within the article or Supplementary Material.

## Supplementary Materials

The following supporting information can be downloaded at: https://www.mdpi.com/article/10.3390/plants15121911/s1, Table S1. Primers used for RT-qPCR validation. Table S2. Program parameters for RT-qPCR. Table S3. Primers used for constructing the plasmids for dual-luciferase assay. Figure S1. Spatiotemporal expression patterns of *SHN1* and *WRI3* during *Arabidopsis* flower development. Figure S2. Phylogenetic tree of PsCER2, PsLTPG1, PsKAS1 and their homologous proteins. Figure S3. Prediction of AtSHN1 binding sites in *CER2* promoters. Figure S4. Predicted AtSHN1 interaction sites in LTPG promoters. Figure S5. Sequence alignment of the *CER2* gene promoter region (from −1300 bp to −22 bp upstream of the start codon) between “Bai Wang Shi Zi” and *Paeonia ostii*. Figure S6. Sequence alignment of the *KAS1* gene promoter region (from −1285 bp to −19 bp upstream of the start codon) between “Bai Wang Shi Zi” and *Paeonia ostii*. Figure S7. Sequence alignment of the *KAS1* gene promoter region (from −736 bp to −1 bp upstream of the start codon) between “Bai Wang Shi Zi” and *Paeonia ostii*. Figure S8. Sequence alignment of the *SHN1* gene coding sequences between “Bai Wang Shi Zi” and *Paeonia ostii*. Figure S9. Sequence alignment of the *WRI3* gene coding sequences between “Bai Wang Shi Zi” and *Paeonia ostii*.
