# Supplementary material for: Transcriptome-Based Identification of AP2/EREBP Genes Regulating Cuticle Formation in Tree Peony ‘Bai Wang Shi Zi’
Source: Plants (Basel). 2026 Jun 20;15(12):1911. doi: 10.3390/plants15121911 (PMC13306563; doi:10.3390/plants15121911)
Supplement: Supplementary file 1 [file plants-15-01911-s001.zip › plants-4316964-supplementary.pdf]

Supplemental Materials

Table S1. Primers used for RT-qPCR validation.

Primers	Sequences	Gene name
PsSHN1-RP-F	5'-GATTCGCCATCCATTACTGA -3'	*PsSHN1*
PsSHN1-RP-R	5'-CAATGTGGGAATTCTCAGTG -3'	
PsWRI3-RP-F	5'-GTTACCAGGCATCGTTGG -3'	*PsWRI3*
PsWRI3-RP-R	5'-CTTGCAACACCTCTGTATTTG -3'	
PsKAS1-RP-F	5'-CGAGTCGTGATAACAGGTATG -3'	*PsKAS1*
PsKAS1-RP-R	5'-CGCTCCACGTCAATCTTC -3'	
PsLTPG1-RP-F	5'-GACGATCGGAGAGAAGTG -3'	*PsLTPG1*
PsLTPG1-RP-R	5'-GGTATGTTCAGGAGCTTGG -3'	
PsCER2-RP-F	5'-CTTGGTCCTGAACTATTCAC -3'	*PsCER2*
PsCER2-RP-R	5'-CTTGGCAGTTATCTGGAAAG -3'	
MDSY-PP2AA3-1	5'-CAGCCAATAAGCGAACAG -3'	*PsPP2AA3*
MDSY-PP2AA3-2	5'-TGTTGAGTCGTTGTGCAG -3'	

Table S2. Program parameters for RT-qPCR.

Step	Temperature (°C)	Time (sec)	Cycles
Initial denaturation	95	30	1
Denaturation	95	5	40
Annealing	56	30	40
Extension	72	30	40

Table S3. Primers used for constructing the plasmids for dual-luciferase assay.

Primer name	Primer sequences	Restriction enzyme
Pos-SHN1-F	CTCTCTAGACAGCAACTCTTCAAAAGACTGAGT	*Xba*I
Pos-SHN1-R	CCTGGGCCCGAAGCACTACCTTTGGAGAGAC	*Apa*I
Pos-WRI3-F	CTGTCTAGACTCAGCGCTCCCATTTCC	*Xba*I
Pos-WRI3-R	CCTGGGCCCCACCATCCTTACCTTCATCTATTCAC	*Apa*I
Pos-CER2-F	GTCGGGCCCCTTCAGCTGTTGGACTCTTCTC	*Apa*I
Pos-CER2-R	CCTCCATGGGATGCACAGCTCACAGGTAG	*Nco*I
Pos-KAS1-F	GTCGGGCCCCCTATGAGTCTGCAATCCTTACG	*Apa*I
Pos-KAS1-R	CCTCCATGGGGAAGAGAGATCGGGGTAAGAAG	*Nco*I
Pos-LTPG1-F	CCTGGGCCCGTGACACGACTTTAGGTTGAGTTG	*Apa*I
Pos-LTPG1-R	CTCGGATCCCGTTTGATTCTTCACACTCACACTC	*Bam*HI

Note: Sequences marked in red are restriction enzyme recognition sites.

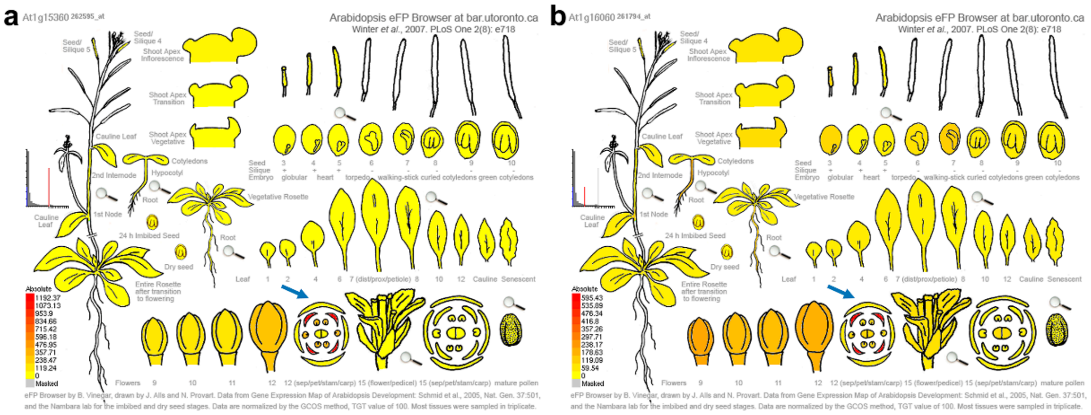

Figure S1. Spatiotemporal expression patterns of *SHN1* and *WRI3* during Arabidopsis flower development. **(a)** Expression profile of *SHN1* (*AT1G15360*), with high expression shown in petals at stage 12. **(b)** Expression profile of *WRI3* (*AT1G16060*), with high expression also shown in petals at stage 12. Blue arrows indicate the developmental stage (stage 12) at which each gene reaches its highest expression level. Data were retrieved from the eFP Browser (http://bar.utoronto.ca/efp/cgi-bin/efpWeb.cgi)

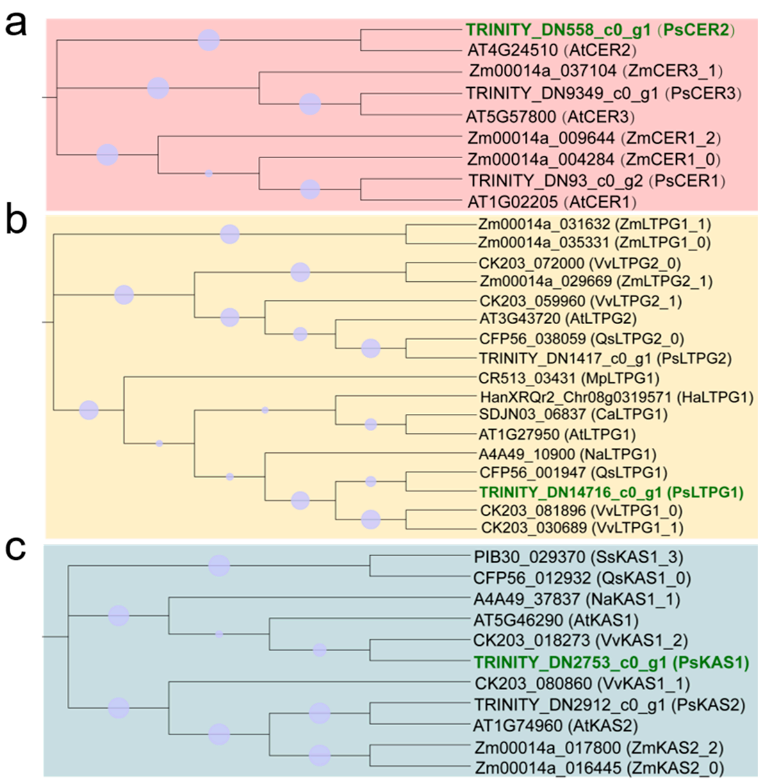

Figure S2. Phylogenetic tree of PsCER2, PsLTPG1, PsKAS1 and their homologous proteins. **(a)** Phylogenetic tree analysis of PsCER2 from 'Bai Wang Shi Zi' and its homologous sequences from Arabidopsis and Zea mays. **(b)** Phylogenetic analysis of PsCER2 from 'Bai Wang Shi Zi' and its homologous sequences from other species, including *Zea mays*, *Vitis vinifera*, *Arabidopsis thaliana*, *Quercus suber*, *Mucuna pruriens*, *Helianthus annuus*, *Cucurbita argyrosperma* and *Nicotiana attenuata*. **(c)** Phylogenetic analysis of PsKAS1 from 'Bai Wang Shi Zi' and its homologous sequences from other species, including *Zea mays*, *Stylosanthes scabra*, *Quercus suber*, *Nicotiana attenuata* and *Vitis vinifera*.

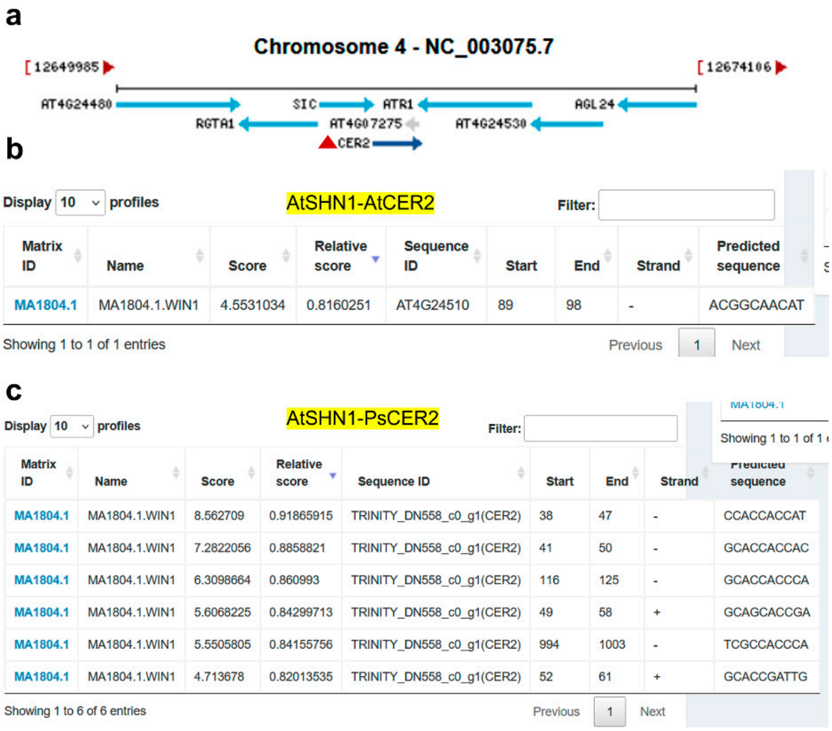

b AtSHN1-AtCER2

Matrix ID	Name	Score	Relative score	Sequence ID	Start	End	Strand	Predicted sequence
MA1804.1	MA1804.1.WIN1	4.5531034	0.8160251	AT4G24510	89	98	-	ACGGCAACAT

Showing 1 to 1 of 1 entries

c AtSHN1-PsCER2

Matrix ID	Name	Score	Relative score	Sequence ID	Start	End	Strand	Predicted sequence
MA1804.1	MA1804.1.WIN1	8.562709	0.91865915	TRINITY_DN558_c0_g1(CER2)	38	47	-	CCACCACCAT
MA1804.1	MA1804.1.WIN1	7.2822056	0.8858821	TRINITY_DN558_c0_g1(CER2)	41	50	-	GCACCACCAC
MA1804.1	MA1804.1.WIN1	6.3098664	0.860993	TRINITY_DN558_c0_g1(CER2)	116	125	-	GCACCACCCA
MA1804.1	MA1804.1.WIN1	5.6068225	0.84299713	TRINITY_DN558_c0_g1(CER2)	49	58	+	GCAGCACCGA
MA1804.1	MA1804.1.WIN1	5.5505805	0.84155756	TRINITY_DN558_c0_g1(CER2)	994	1003	-	TCGCCACCCA
MA1804.1	MA1804.1.WIN1	4.713678	0.82013535	TRINITY_DN558_c0_g1(CER2)	52	61	+	GCACCGATTG

Showing 1 to 6 of 6 entries

Figure S3. Prediction of AtSHN1 binding sites in *CER2* promoters. **(a)** Chromosomal location of the Arabidopsis *CER2* gene annotated by the NCBI database. **(b)** Predicted binding sites of AtSHN1 within the *AtCER2* promoter. **(c)** Predicted binding sites of AtSHN1 within the *PsCER2* promoter.

(Note: Sequences from NCBI; prediction using JASPAR 2026.)

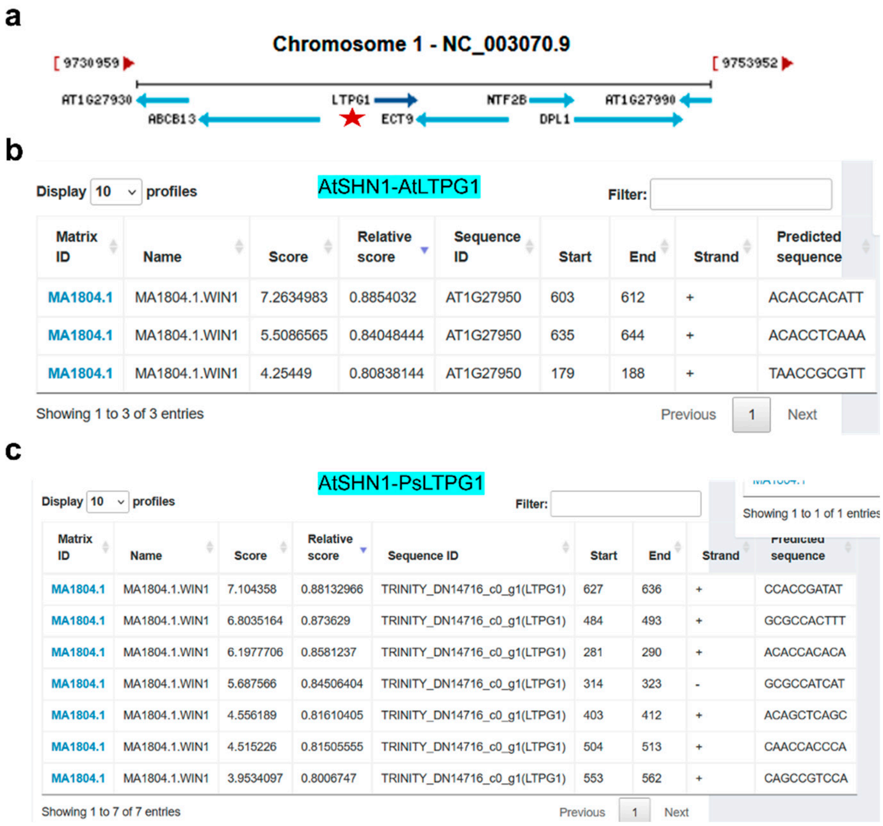

b AtSHN1-AtLTPG1

Matrix ID	Name	Score	Relative score	Sequence ID	Start	End	Strand	Predicted sequence
MA1804.1	MA1804.1.WIN1	7.2634983	0.8854032	AT1G27950	603	612	+	ACACCACATT
MA1804.1	MA1804.1.WIN1	5.5086565	0.84048444	AT1G27950	635	644	+	ACACCTCAAA
MA1804.1	MA1804.1.WIN1	4.25449	0.80838144	AT1G27950	179	188	+	TAACCGCGTT

Showing 1 to 3 of 3 entries — Previous 1 Next

c AtSHN1-PsLTPG1

Matrix ID	Name	Score	Relative score	Sequence ID	Start	End	Strand	Predicted sequence
MA1804.1	MA1804.1.WIN1	7.104358	0.88132966	TRINITY_DN14716_c0_g1(LTPG1)	627	636	+	CCACCGATAT
MA1804.1	MA1804.1.WIN1	6.8035164	0.873629	TRINITY_DN14716_c0_g1(LTPG1)	484	493	+	GCGCCACTTT
MA1804.1	MA1804.1.WIN1	6.1977706	0.8581237	TRINITY_DN14716_c0_g1(LTPG1)	281	290	+	ACACCACACA
MA1804.1	MA1804.1.WIN1	5.687566	0.84506404	TRINITY_DN14716_c0_g1(LTPG1)	314	323	-	GCGCCATCAT
MA1804.1	MA1804.1.WIN1	4.556189	0.81610405	TRINITY_DN14716_c0_g1(LTPG1)	403	412	+	ACAGCTCAGC
MA1804.1	MA1804.1.WIN1	4.515226	0.81505555	TRINITY_DN14716_c0_g1(LTPG1)	504	513	+	CAACCACCCA
MA1804.1	MA1804.1.WIN1	3.9534097	0.8006747	TRINITY_DN14716_c0_g1(LTPG1)	553	562	+	CAGCCGTCCA

Showing 1 to 7 of 7 entries — Previous 1 Next

Figure S4. Predicted AtSHN1 interaction sites in LTPG promoters. **(a)** Chromosomal localization of *AtLTPG1* based on NCBI annotation. **(b)** Predicted AtSHN1-binding motifs in the *AtLTPG1* promoter. **(c)** Predicted AtSHN1-binding motifs in the *PsLTPG1* promoter.
(Note: NCBI source sequences; JASPAR 2026 for prediction.)

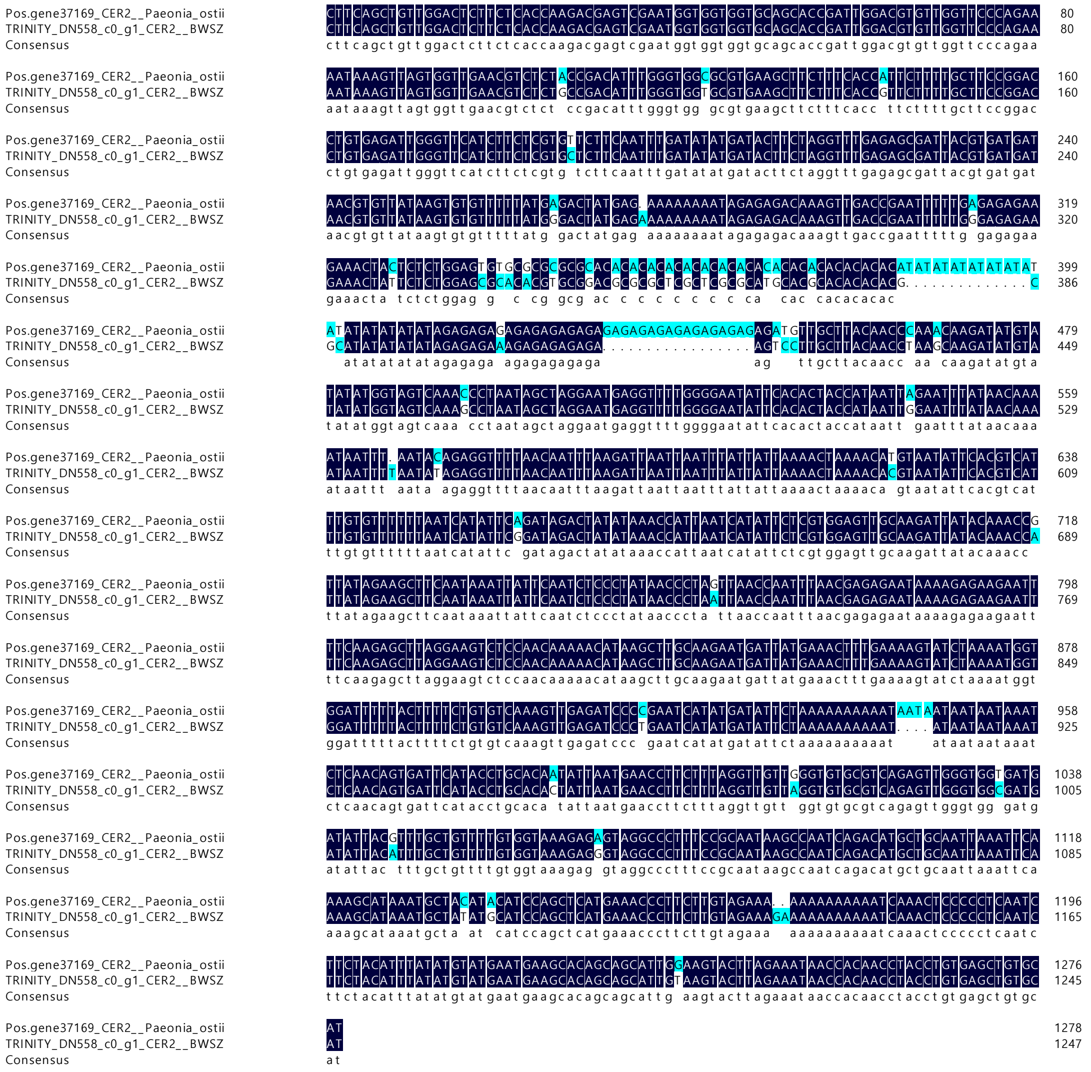

Figure S5. Sequence alignment of the *CER2* gene promoter region (from -1300 bp to -22 bp upstream of the start codon) between "Bai Wang Shi Zi" and *Paeonia ostii*. The results show that the promoter sequence identity between the two cultivars is 93.06%.

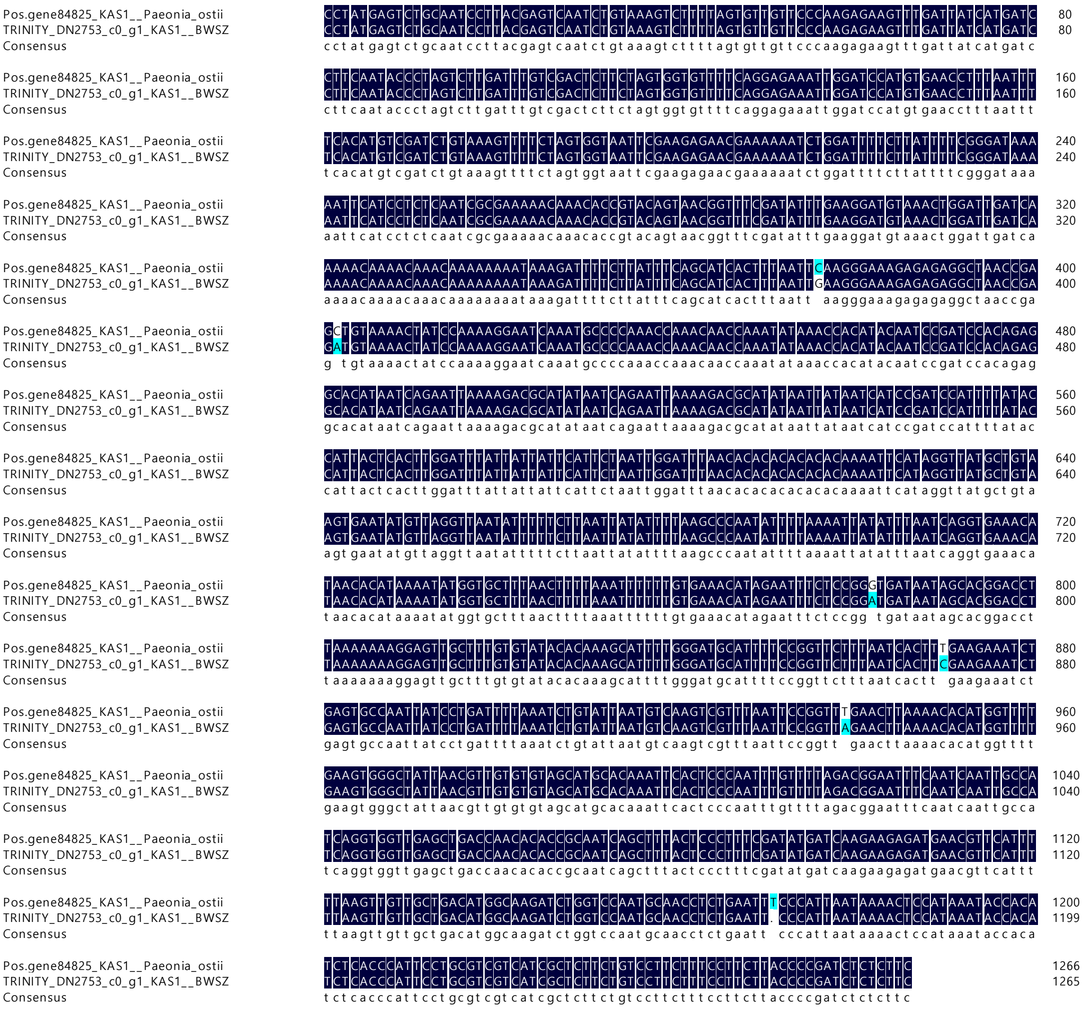

Figure S6. Sequence alignment of the *KAS1* gene promoter region (from -1285 bp to -19 bp upstream of the start codon) between “Bai Wang Shi Zi” and *Paeonia ostii*. The results show that the promoter sequence identity between the two cultivars is 99.93%.

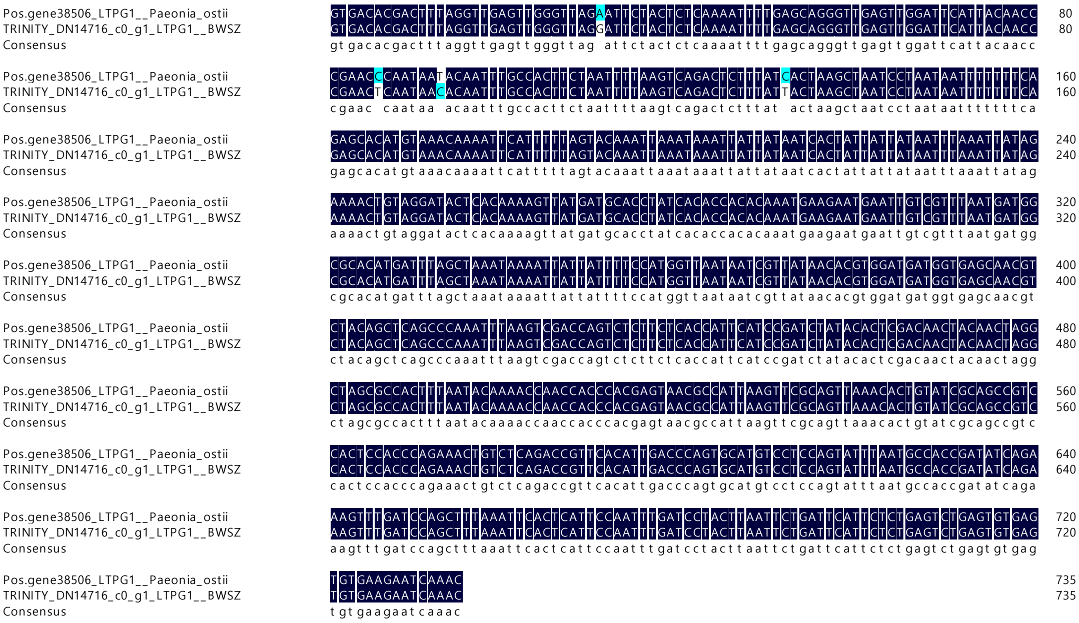

Figure S7. Sequence alignment of the *KAS1* gene promoter region (from -736 bp to -1 bp upstream of the start codon) between “Bai Wang Shi Zi” and *Paeonia ostii*. The results show that the promoter sequence identity between the two cultivars is 99.46%.

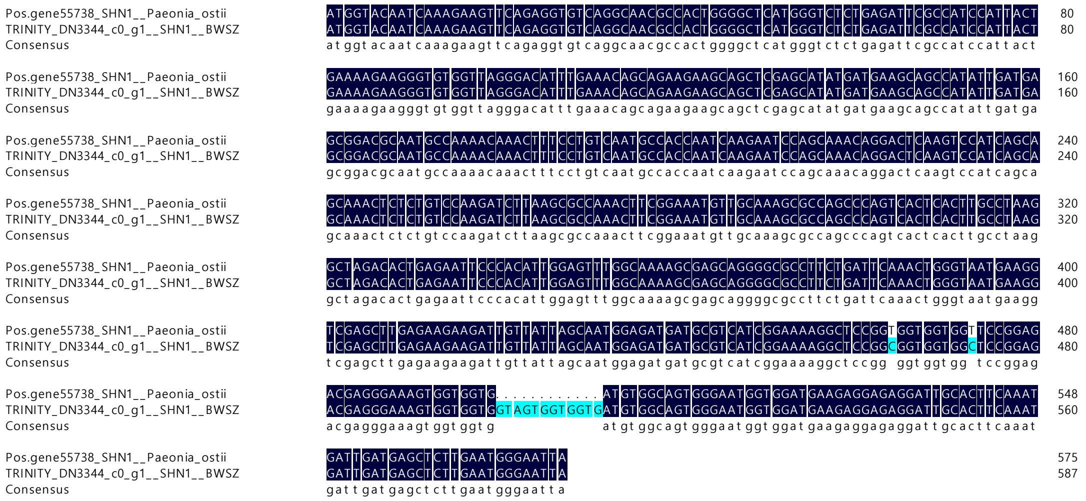

Figure S8. Sequence alignment of the *SHN1* gene coding sequences between “Bai Wang Shi Zi” and *Paeonia ostii*. The results show that the coding sequence identity between the two cultivars is 97.62%.

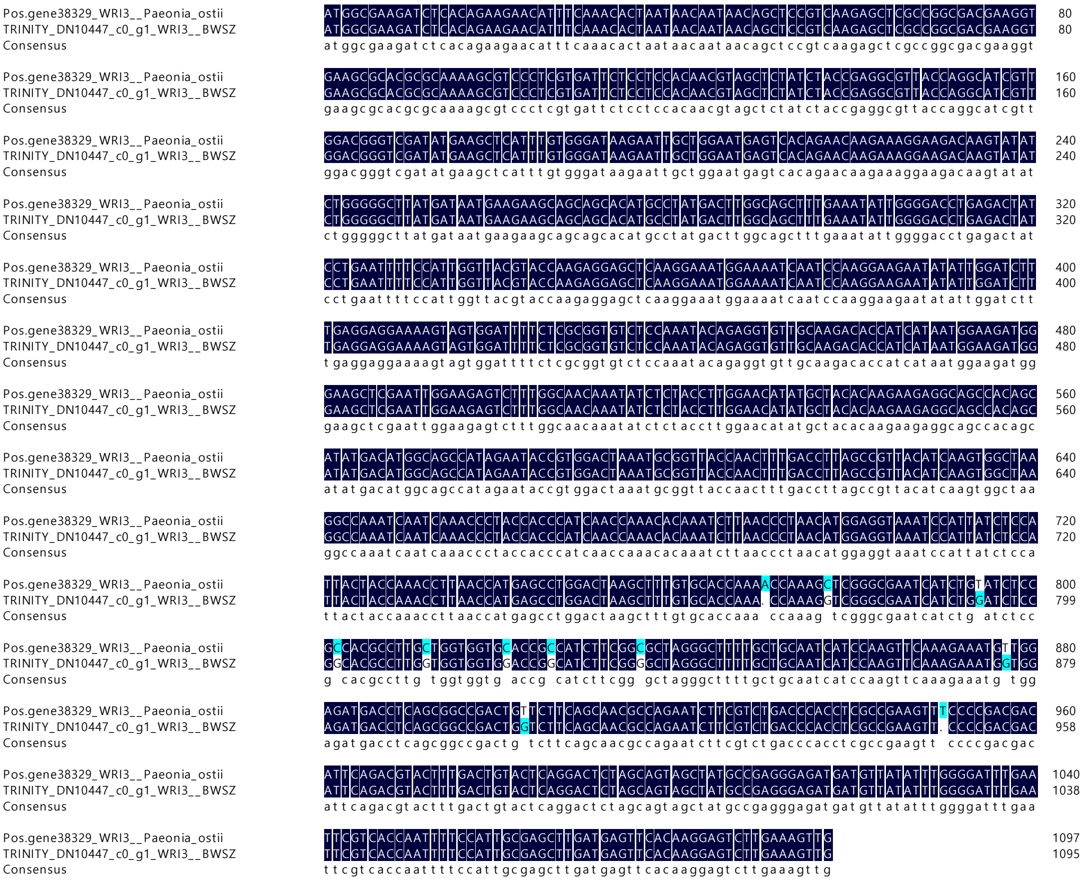

Figure S9. Sequence alignment of the *WRI3* gene coding sequences between "Bai Wang Shi Zi" and *Paeonia ostii*. The results show that the coding sequence identity between the two cultivars is 99.0%.
